# Proanthocyanidins inhibit CYP1B1 through mixed-type kinetics and stable binding in molecular dynamics simulations

**DOI:** 10.1038/s41598-025-12781-2

**Published:** 2025-08-29

**Authors:** Liwei Jia, Lei Zhou, Shujun Zou, Yang Xu, Xin Meng

**Affiliations:** School of Pharmacy, Heilongjiang University of Chinese Medicine, No.24 Heping Road, Harbin, 150040 People’s Republic of China

**Keywords:** Proanthocyanidin, CYP1B1, Inhibition kinetics, Molecular dynamics, ADMET, Biocatalysis, Breast cancer

## Abstract

Cytochrome P450 1B1 (CYP1B1) is a heme-containing enzyme involved in procarcinogen activation and estrogen metabolism, contributing to tumor progression. This study investigates the inhibitory effects of proanthocyanidin (PA) on CYP1B1-catalyzed reactions and its underlying mechanisms. Enzyme kinetics revealed that PA exerts mixed-type inhibition with an IC₅₀ of 2.53 ± 0.01 μM. Molecular docking demonstrated that PA binds to key residues (Phe231, Gly329, Ala330, Asn228, Asn265) and the heme cofactor through hydrogen bonding and π–π stacking, interfering with substrate binding and electron transfer. Molecular dynamics simulations over 200 ns confirmed the stability of the PA-CYP1B1 complex. To validate the stability and inhibitory relevance of the simulation results, berberine, a known CYP1B1 inhibitor, was used as a positive control in parallel analyses. In silico ADMET prediction indicated high intestinal absorption and a favorable safety profile, with no significant CYP inhibition or mutagenicity. However, low membrane permeability and multiple drug-likeness violations suggest limited oral bioavailability. These findings support the potential of PA as a natural CYP1B1 inhibitor for cancer prevention and treatment. Further structural optimization or formulation strategies may enhance its pharmacokinetic properties and clinical applicability.

## Introduction

Cytochrome P450 (CYP) enzymes are essential hemoproteins involved in the metabolism of a wide array of endogenous substrates and xenobiotics, including drugs, environmental toxins, procarcinogens, and steroid hormones^1,2^. Among the various CYP isoforms, cytochrome P450 1B1 (CYP1B1) has garnered significant attention due to its dual role in both normal physiological functions and pathological conditions. CYP1B1 is implicated in the metabolic activation of polycyclic aromatic hydrocarbons (PAHs) and other environmental pollutants, converting them into reactive intermediates capable of binding to DNA and triggering mutagenesis^3–5^. Notably, CYP1B1 is also involved in the metabolism of 17-β-estradiol to 4-hydroxy-17-β-estradiol and subsequently to estradiol-3,4-quinones, highly reactive species that can form DNA adducts and promote estrogen-dependent carcinogenesis^6–8^. These metabolic transformations increase the risk of hormone-related cancers, including breast, ovarian, and endometrial cancers.

Overexpression of CYP1B1 in various tumors compared to adjacent normal tissues has been consistently documented^9–11^. This overexpression correlates with tumor aggressiveness, chemoresistance, and poor patient prognosis. For example, CYP1B1 mediated metabolism can alter the intracellular disposition of chemotherapeutic drugs, conferring resistance to commonly used anticancer agents. Given its tumor-specific expression pattern and role in carcinogen bioactivation, CYP1B1 has emerged as a promising target for cancer chemoprevention and therapy. Inhibiting CYP1B1 could prevent the formation of harmful intermediates, slow down cancer progression, sensitize tumors to chemotherapy, and improve clinical outcomes.

Although synthetic CYP1B1 inhibitors have shown promise, their clinical translation often encounters challenges. Synthetic compounds can have limited bioavailability, off-target effects, and potential toxicity^12–14^. This situation underscores the need for safer, more accessible, and broadly effective agents. In recent years, naturally occurring phytochemicals have gained popularity as potential CYP1B1 inhibitors due to their favorable safety profiles, abundant availability, and pleiotropic biological activities^15–17^. Among these natural compounds, polyphenols have emerged as prime candidates, as they are commonly found in fruits, vegetables, tea, wine, and various medicinal plants.

Proanthocyanidins (PAs) are a class of oligomeric flavan-3-ols widely distributed in nature, present in grapes, berries, cocoa, pine bark, and numerous traditional herbal preparations^18,19^. They are known for their potent antioxidant capabilities and wide-ranging health benefits. Extensive research on PAs has highlighted their roles in inhibiting tumor cell proliferation, suppressing angiogenesis and metastasis, modulating inflammatory responses, and inducing apoptosis in various cancer cell models^20–22^. Although some studies have suggested that PAs can affect intracellular signaling pathways and modulate drug-metabolizing enzymes, whether they directly inhibit CYP1B1-mediated catalysis remains unclear.

Elucidating whether PAs can suppress CYP1B1’s catalytic function will provide valuable insights into one of the possible molecular mechanisms underlying their anticancer and chemopreventive actions. If PAs inhibit CYP1B1 activity, they may reduce the formation of DNA-reactive estrogen metabolites and limit the activation of environmental procarcinogens, thereby lowering the risk of cancer initiation and progression. Such a discovery would also encourage the exploration of PA-rich diets or dietary supplements as adjunct strategies in cancer prevention, as well as inspire the synthesis of PA-based analogs with enhanced potency and specificity.

In this study, we sought to address this knowledge gap by investigating the inhibitory effect of proanthocyanidin (PA) on CYP1B1-catalyzed ethoxyresorufin O-deethylation, a well-established assay for probing CYP1-family activity^23^. We determined the IC_50_ value, established the inhibition kinetics (V_max_, K_m_), and deduced the inhibition type (mixed-type). Additionally, we employed molecular docking simulations to explore the structural basis of PA-CYP1B1 interactions and to identify key amino acid residues critical for inhibitor binding. By comparing PA’s binding profile with that of known CYP1B1 inhibitors, we aimed to discover shared interaction motifs and mechanistic commonalities. To further evaluate the pharmacokinetic characteristics and molecular stability of the PA-CYP1B1 interaction, we integrated in silico ADMET predictions and molecular dynamics (MD).

This integrative approach-combining enzymatic kinetics, computational modeling, and structure–activity relationship (SAR) considerations-provides a comprehensive understanding of how PAs exert their inhibitory effect on CYP1B1. Our findings may guide future endeavors to optimize PA derivatives for improved inhibition and selectivity. Such advances could contribute to innovative prevention strategies, reducing the burden of hormone-related and environmentally induced cancers without the side effects often associated with synthetic inhibitors.

## Materials and methods

### Chemicals and reagents

Proanthocyanidin (PA) was purchased from Shanghai Yuanye Bio-Technology Co. (Shanghai, China) and confirmed for purity via standard spectroscopic methods (e.g., UV-Vis, HPLC). NADPH (Roche Diagnostics GmbH, Germany) served as the reducing cofactor for CYP-catalyzed reactions. Recombinant human CYP1B1 and ethoxyresorufin (substrate) were obtained from Discovery Labware, Inc. and AAT Bioquest, respectively. Resorufin, the fluorescent product of O-deethylation, was from Shanghai Yuanye Bio-Technology Co. All other reagents, including phosphate buffer, MgCl_2_, and acetonitrile, were of analytical grade (Xilong Scientific Co., Ltd., China).

### Enzyme inhibition assay

The inhibitory effect of PA on CYP1B1-mediated O-deethylation of ethoxyresorufin was measured according to a modified literature procedure^24^. A reaction mixture (200 μL) contained 0.1 M phosphate buffer (pH 7.4), 20 mM MgCl_2_, 4 mM NADH, 4 mM NADPH, and 1 pmol CYP1B1. PA was added at varying concentrations (0, 1, 2, 5, 10 μM), followed by a 10-min preincubation at 37 °C to ensure equilibration. The reaction was initiated by adding 20 μL ethoxyresorufin and incubated for 20 min. Afterward, ice-cold acetonitrile was used to quench the reaction. The formation of resorufin was detected using a SpectraMax M2 microplate reader (Molecular Devices, USA) at excitation and emission wavelengths of 530 and 590 nm, respectively. IC_50_ values were derived from nonlinear least-squares regression analysis using Origin software^25^.

### Kinetic analysis

To determine the type of inhibition, we varied the substrate concentration (0, 0.02, 0.05, 0.10, 0.20, 0.50, 0.80 mM ethoxyresorufin) in the absence or presence of PA at its IC_50_ (2.53 μM). After a 10-min preincubation, CYP1B1 was introduced, and the reaction was allowed to proceed for 20 min. The reaction was quenched with ice-cold acetonitrile, and resorufin formation was quantified fluorometrically. Michaelis–Menten kinetics were applied to determine V_max_ and K_m_. Lineweaver–Burk double-reciprocal plots were constructed to identify the inhibition type. Mixed-type inhibition is indicated when both K_m_ and V_max_ are affected, and the plot lines intersect in the second quadrant.

### Molecular docking

The crystal structure of human CYP1B1 (PDB ID: 6IQ5) was downloaded from the RCSB Protein Data Bank^26^. Using Discovery Studio (DS), crystallographic water and ligands were removed, hydrogen atoms were added, and the protein structure was minimized. The active site was identified with the “From Receptor Cavities” module, focusing on residues near the heme cofactor.

The structure of PA was retrieved from the PubChem database and prepared using Open Babel and DS’s “Prepare Ligands” module. As references, four known CYP1B1 inhibitors (berberine, resveratrol, 3-hydroxyflavone, 2-phenylchromone) were docked to CYP1B1 to identify critical binding residues. PA was then docked into the CYP1B1 active site using CDOCKER, a CHARMm-based algorithm. The best-docked poses were analyzed for hydrogen bonding, π–π stacking, and hydrophobic interactions. A 2D interaction diagram was generated to highlight key receptor-ligand contacts.

### Molecular dynamics simulation

All molecular dynamics (MD) simulations were conducted using GROMACS 2020.6. The protein–ligand complex was prepared for a 200 ns simulation. The AMBER99SB-ILDN force field was used to generate the topology of the CYP1B1 protein, while the topology of the proanthocyanidin ligand was constructed using the General Amber Force Field (GAFF) via AmberTools20.

The complex was solvated in a truncated octahedral Transferable Intermolecular Potential with Points (TIP3P) water box with a 1.0 nm padding distance from the complex to the box edges. Appropriate numbers of Na^+^ and Cl⁻ ions were added to neutralize the system’s net charge. Energy minimization was performed using 2,500 steps of the steepest descent algorithm followed by 2,500 steps of conjugate gradient minimization to remove steric clashes and optimize the geometry. Subsequently, the system was equilibrated in two phases: a 100 ps NVT (constant number of particles, volume, and temperature) ensemble followed by a 100 ps NPT (constant number of particles, pressure, and temperature) ensemble, maintaining the temperature at 300 K and pressure at 1 bar using the V-rescale thermostat and Parrinello-Rahman barostat, respectively.

Finally, a 200 ns production MD simulation was carried out under periodic boundary conditions with a time step of 2 fs. The trajectory was saved every 10 ps for further analysis. In addition, to serve as a positive control, a separate 200 ns MD simulation was conducted for the CYP1B1–berberine complex using the same force field parameters, solvation model, and simulation protocol as the proanthocyanidin system. This parallel simulation allowed for direct comparative analysis of dynamic stability, protein flexibility, and ligand binding behavior between the two systems. Post-simulation, key parameters including root mean square deviation (RMSD), root mean square fluctuation (RMSF), radius of gyration (Rg), and solvent-accessible surface area (SASA) were computed to assess the structural stability and conformational dynamics of the CYP1B1-proanthocyanidin complex.

### ADMET prediction

In silico absorption, distribution, metabolism, excretion, and toxicity (ADMET) properties of proanthocyanidin were predicted using ADMETlab 2.0 (https://admetmesh.scbdd.com), an integrated online platform for comprehensive ADMET evaluation. The chemical structure of proanthocyanidin (CAS No. 4852-22-6) was retrieved from the PubChem database in SDF format. The SDF file was then converted to SMILES notation using the built-in conversion tool on the ADMETlab 2.0 website.

The resulting SMILES string was used as input for ADMET prediction, covering multiple endpoints across five pharmacokinetic categories: absorption (e.g., Human Intestinal Absorption, Caco-2 permeability), distribution (e.g., BBB penetration), metabolism (e.g., CYP3A4 inhibition), excretion (e.g., clearance), and toxicity (e.g., AMES, hepatotoxicity, carcinogenicity). All predictions were performed using the platform’s default model parameters and statistical thresholds.

### Statistical analysis

All experiments were performed in quadruplicate. Data are expressed as mean ± standard deviation (SD). Statistical significance was assessed using one-way ANOVA followed by Dunnett’s multiple comparison test, with p < 0.05 considered significant.

## Results

### Inhibitory effect of PA on CYP1B1 activity

**Fig. 1:**
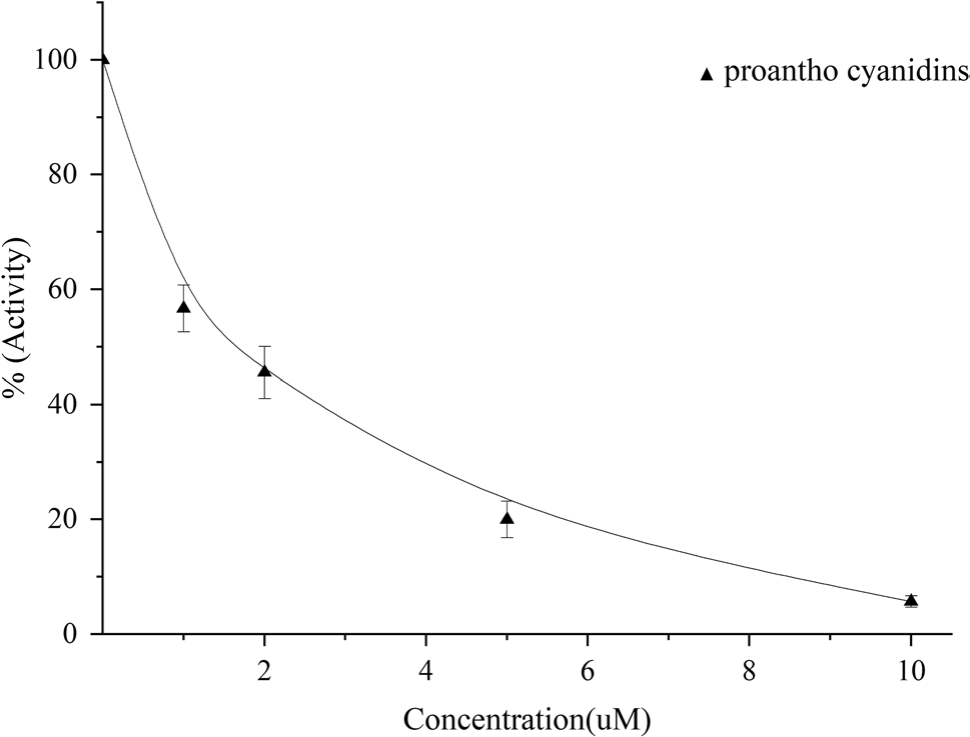
Dose–response curve of proanthocyanidin (PA) inhibiting CYP1B1 activity.

PA exhibited a clear, dose-dependent inhibition of CYP1B1-catalyzed ethoxyresorufin O-deethylation (Fig. 1). Even at low micromolar concentrations, PA significantly suppressed resorufin formation, indicating potent enzyme inhibition. As PA concentration increased, the enzyme’s activity declined proportionally. The IC_50_ value of 2.53 ± 0.01 μM confirmed that PA is a relatively strong CYP1B1 inhibitor, comparable to or better than some previously reported synthetic inhibitors.

### Enzyme kinetics

**Fig. 2:**
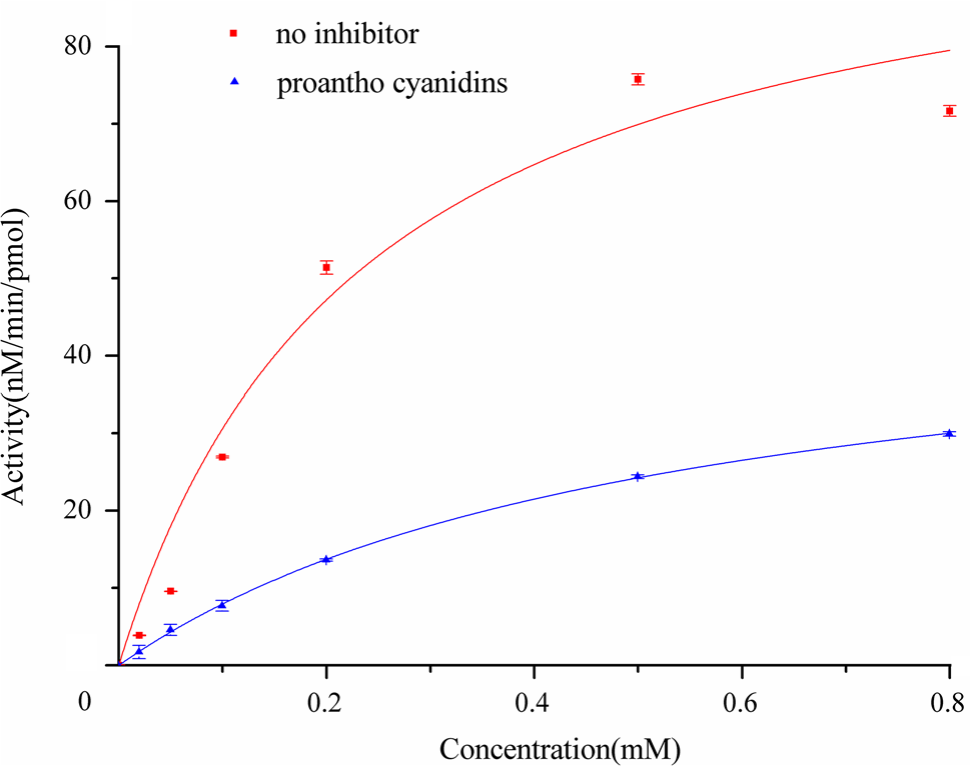
Michaelis–Menten kinetics of ethoxyresorufin catalysis by CYP1B1 with and without PA.

Michaelis–Menten kinetics revealed that in the absence of PA, CYP1B1 displayed a V_max_ of 102.98 ± 12.91 nM/min/pmol CYP1B1 and a K_m_ of 0.236 ± 0.074 mM (Fig. 2). These parameters indicate a robust catalytic turnover and moderate substrate affinity under baseline conditions. The addition of PA at its IC_50_ reduced V_max_ to 49.79 ± 0.88 nM/min/pmol CYP1B1—approximately a 50% decrease-while increasing K_m_ to 0.525 ± 0.02 mM, suggesting diminished substrate affinity.

**Fig. 3:**
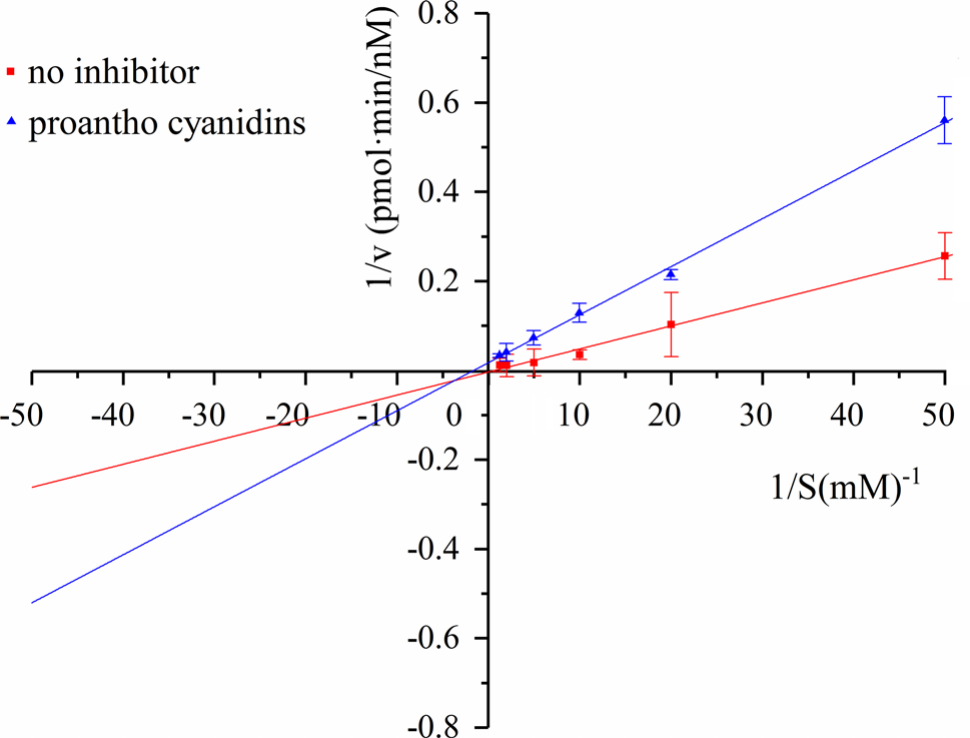
Lineweaver-Burk plot showing mixed-type inhibition of CYP1B1 by PA.

Lineweaver–Burk analysis confirmed a mixed-type inhibition pattern (Fig. 3). The altered slope and intercept, with intersection in the second quadrant, are hallmarks of mixed-type inhibition, where the inhibitor can bind to both free enzyme and the enzyme–substrate complex. Thus, PA affects multiple steps of the catalytic cycle, limiting both substrate binding and product formation.

Molecular Docking and Interaction with Key Residues.

### Molecular docking and interaction with key residues

**Fig. 4:**
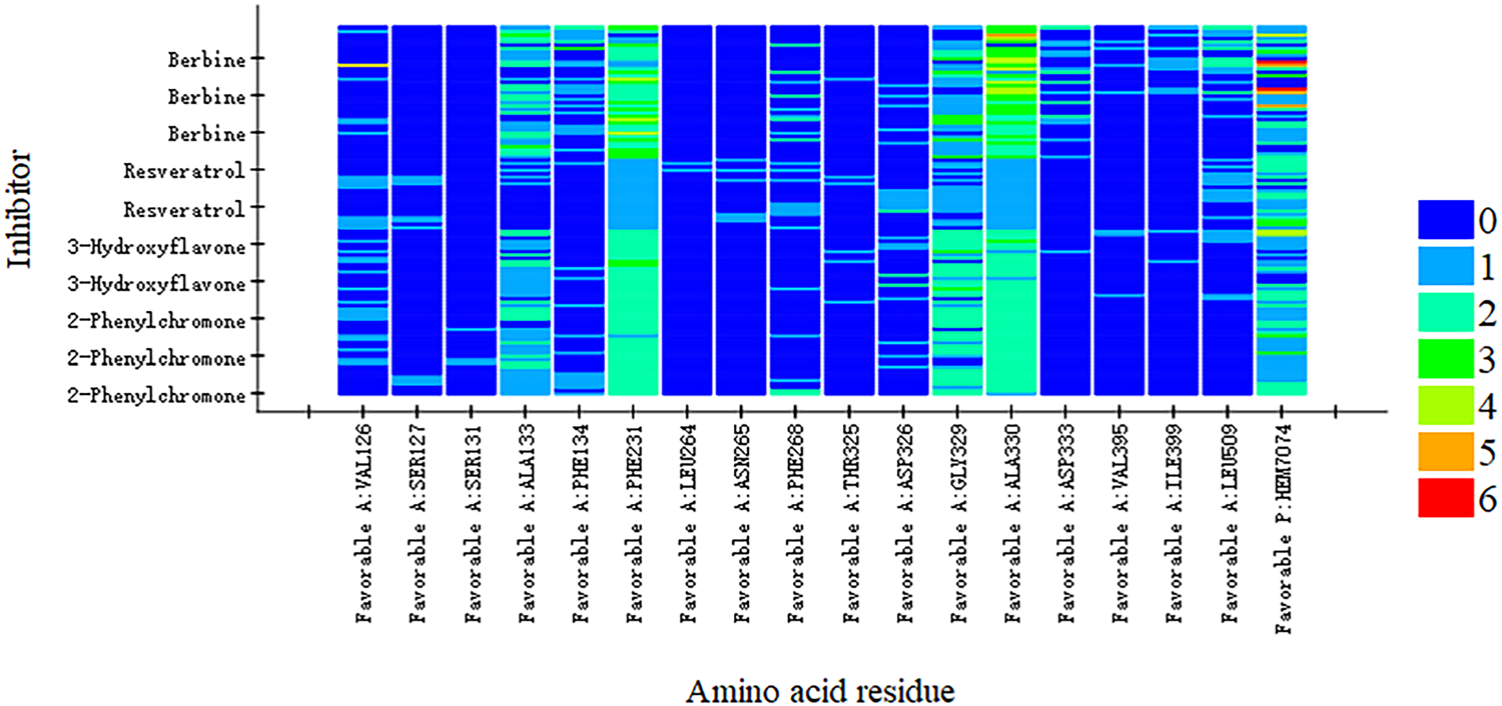
Heatmap of interaction between CYP1B1 key residues and known inhibitors.

Molecular docking provided a structural basis for PA’s inhibitory activity. By comparing known inhibitors (berberine, resveratrol, 3-hydroxyflavone, and 2-phenylchromone), we identified residues frequently involved in inhibitor binding, including Phe231, Gly329, and Ala330 (Fig. 4).

**Fig. 5:**
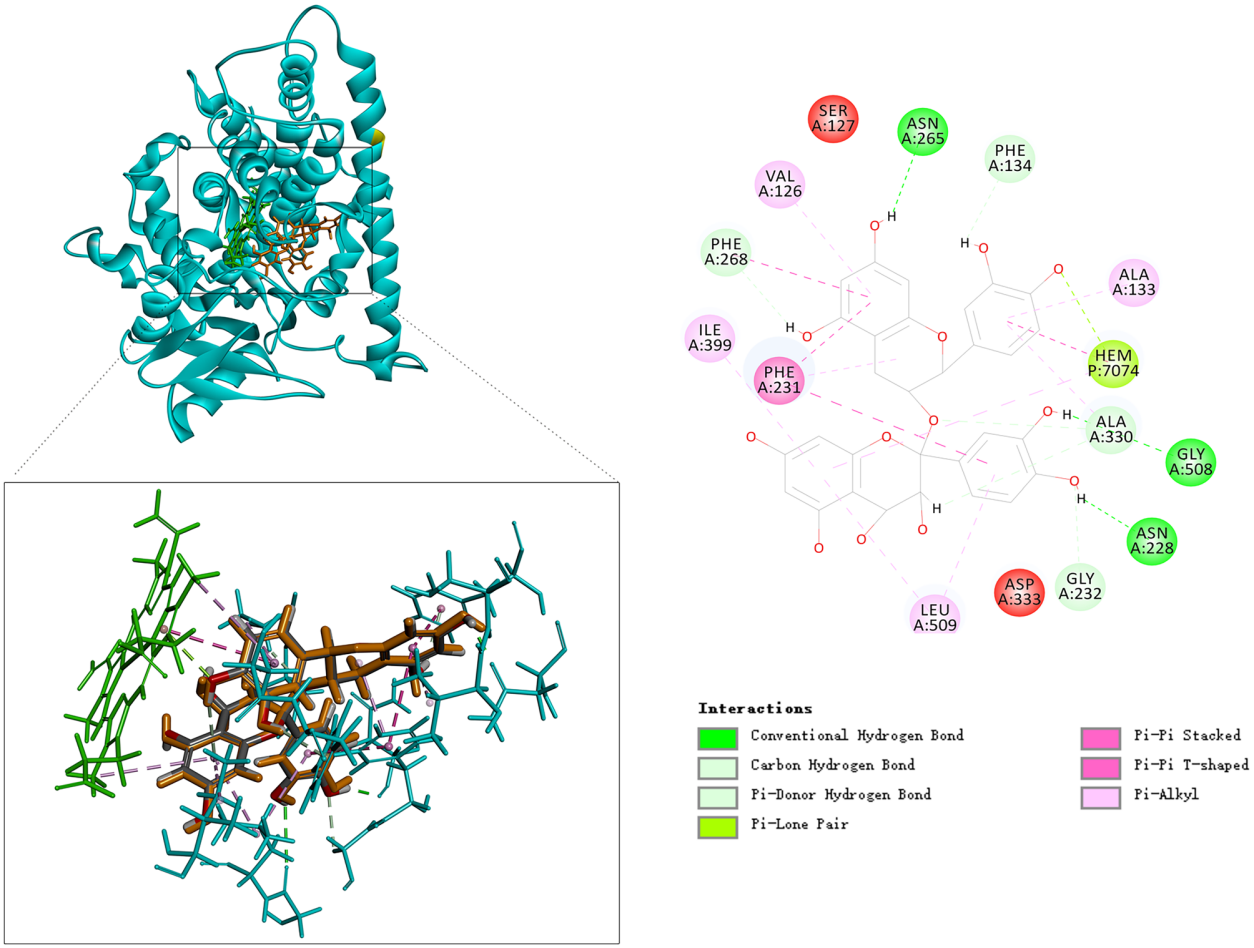
Docking pose of proanthocyanidin in CYP1B1 active site.

PA’s best-docked pose showed hydrogen bond interactions with Asn228 and Asn265, and π-π stacking with Phe231 and the heme cofactor (Fig. 5). Additional stabilizing contacts involved Ala133 and Gly508. The combination of hydrogen bonding, π–π interactions, and hydrophobic contacts strongly anchors PA within the CYP1B1 active site, likely hindering substrate approach, orientation, and subsequent electron transfer required for catalysis.

### RMSD analysis of CYP1B1 complexes with proanthocyanidin and berberine

**Fig. 6:**
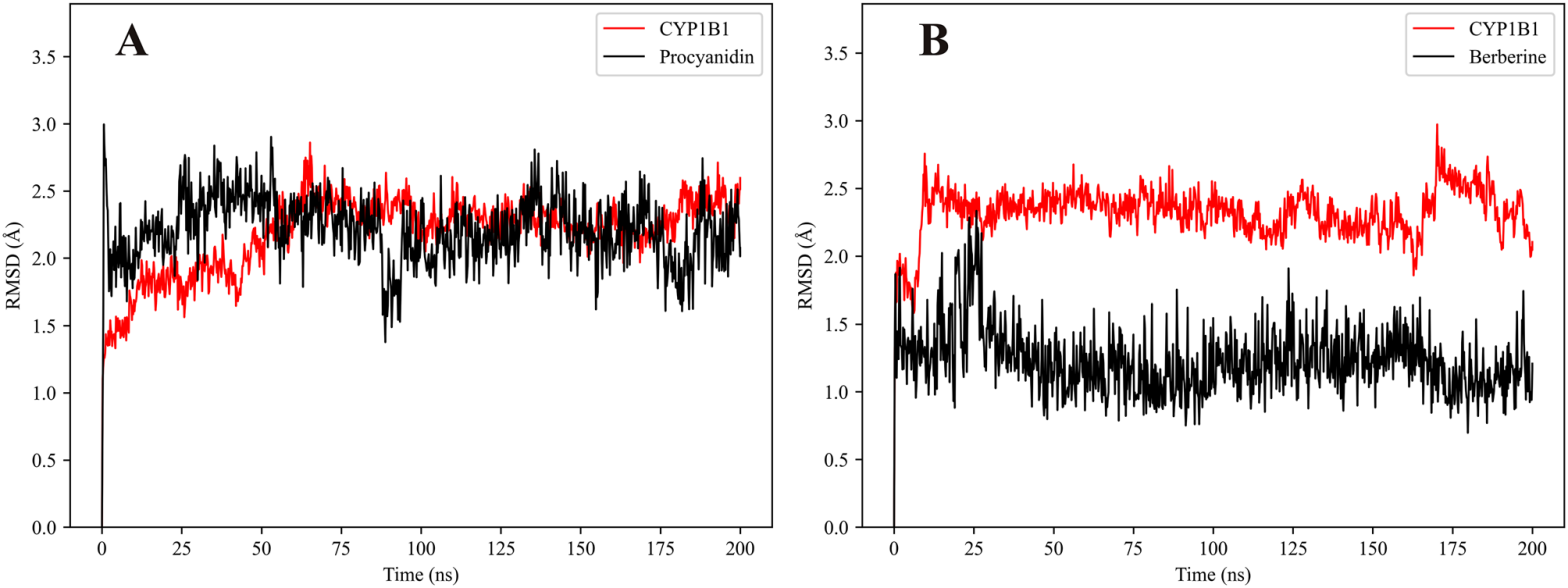
RMSD profiles of CYP1B1 in complex with proanthocyanidin (**A**) and berberine (**B**) during 200 ns molecular dynamics simulations.

The root mean square deviation (RMSD) is a key metric to evaluate the structural stability and convergence of molecular dynamics (MD) simulations. As shown in Fig. 6A, the backbone RMSD of the CYP1B1 protein in the proanthocyanidin-bound complex (red line) increased rapidly during the initial 10 ns of simulation and gradually stabilized after approximately 40 ns, indicating that the protein system reached equilibrium and remained structurally consistent throughout the remainder of the 200 ns trajectory. In contrast, the RMSD of the proanthocyanidin ligand (black line) showed higher fluctuation during the initial 50 ns, after which the deviation stabilized around 1 Å. This suggests that the ligand adjusted its conformation within the binding pocket before achieving a stable interaction with the protein.

To validate and compare the binding stability, we additionally performed a 200 ns MD simulation using berberine as a known CYP1B1 inhibitor (Fig. 6B). The RMSD of the CYP1B1 backbone in this complex also reached equilibrium within 20 ns and maintained a stable fluctuation range between 1.5 and 2.0 Å, indicating overall structural stability of the protein. Notably, the RMSD of berberine was consistently low (~ 1.3 Å) and quickly stabilized, reflecting rapid convergence and a more rigid binding conformation compared to proanthocyanidin. These results suggest that berberine achieves rapid stabilization, while proanthocyanidin undergoes a longer adaptation process before attaining stable binding, while proanthocyanidin may require conformational adaptation to achieve a comparable level of interaction stability.

### RMSF analysis of CYP1B1

To evaluate how different ligands influence the local flexibility of CYP1B1, we performed root mean square fluctuation (RMSF) analysis of the protein backbone residues over the 200 ns molecular dynamics simulation.

**Fig. 7:**
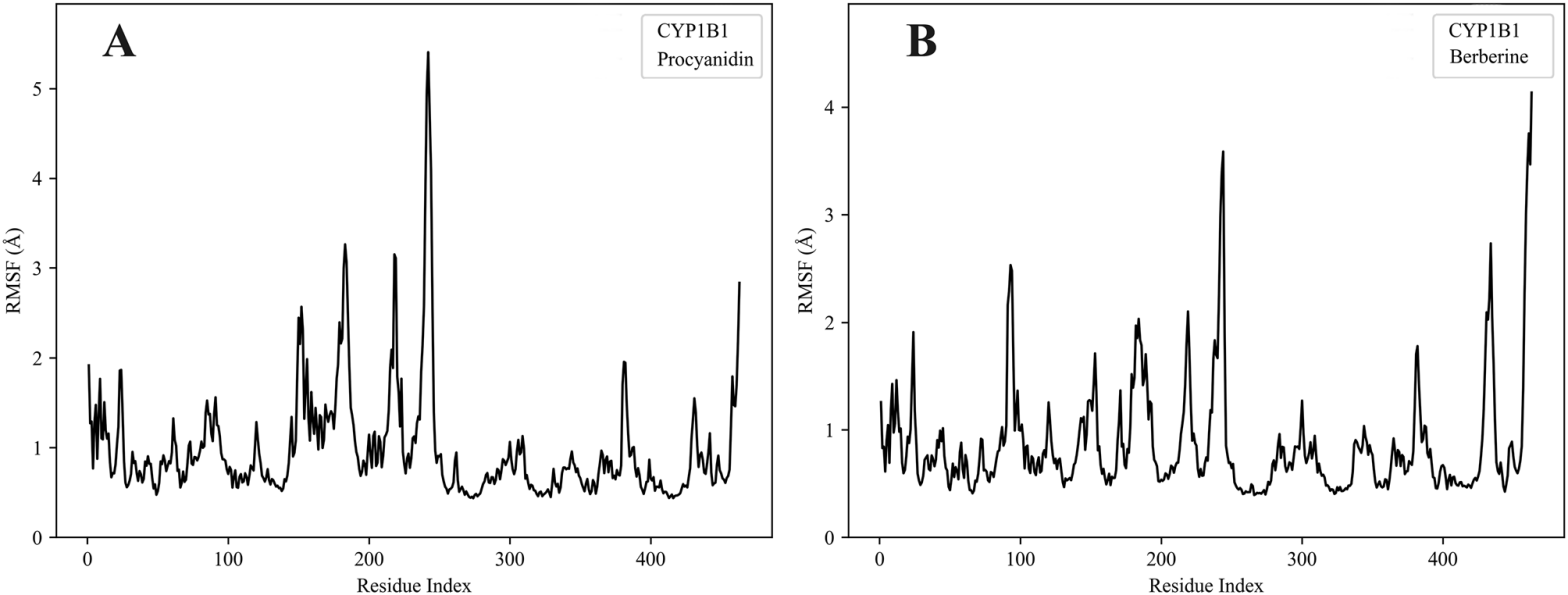
RMSF comparison of CYP1B1 residues over 200 ns MD simulations in complex with proanthocyanidin and berberine. (**A**) RMSF of CYP1B1 in complex with proanthocyanidin. (**B**) RMSF of CYP1B1 in complex with berberine.

As shown in Fig. 7A, when proanthocyanidin is bound to CYP1B1, the majority of protein residues display RMSF values below 1.5 Å, indicating overall structural stability. Pronounced fluctuations are observed in three regions: residues 150–180, around residue 250, and the C-terminal, with peak values approaching 5.0 Å. These fluctuations likely correspond to flexible loop or coil regions that are naturally mobile.

In Fig. 7B, a comparable fluctuation pattern is observed in the berberine-bound CYP1B1 complex. Although some regions—particularly the loops and termini—exhibit slightly lower RMSF values than in the proanthocyanidin-bound form, both systems maintain an overall stable conformation throughout the simulation.

These findings indicate that proanthocyanidin, like the known CYP1B1 inhibitor berberine, is capable of forming a stable complex with the enzyme without causing significant structural destabilization. The similar fluctuation profiles reinforce the potential of proanthocyanidin as a functional CYP1B1 inhibitor.

### RMSF analysis of proanthocyanidins and berberine

**Fig. 8:**
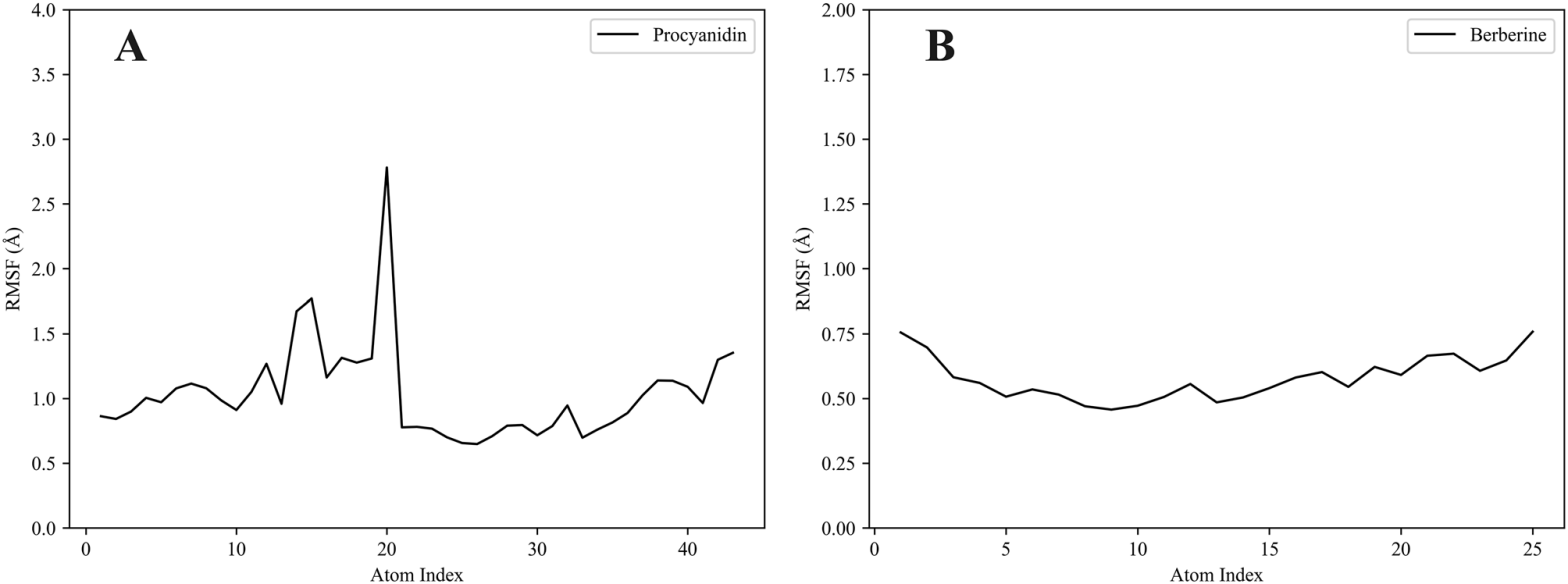
RMSF of ligand atoms during 200 ns MD simulation within the CYP1B1 binding site. (**A**) RMSF of proanthocyanidin atoms in the CYP1B1 binding pocket. (**B**) RMSF of berberine atoms in the CYP1B1 binding pocket.

To further examine the dynamic behavior of ligands within the CYP1B1 binding pocket, root mean square fluctuation (RMSF) analysis was conducted for both proanthocyanidin and berberine over the 200 ns molecular dynamics (MD) simulations. The RMSF plots are presented in Fig. 8A,B.

As shown in Fig. 8A, the majority of proanthocyanidin atoms exhibited fluctuations below 1.5 Å, suggesting stable interactions with the surrounding protein residues. A few atoms—particularly those located in the central region of the molecule—showed higher fluctuations exceeding 2.0 Å, with a peak approaching 2.8 Å. This moderate flexibility is likely due to conformational adjustments of peripheral functional groups, which may facilitate better accommodation within the enzyme’s binding pocket.

In contrast, Fig. 8B shows that berberine atoms displayed overall lower RMSF values, remaining well below 2.0 Å across the entire structure. This indicates that berberine adopts a more rigid and stable binding conformation.

These results suggest that while proanthocyanidin retains structural adaptability necessary for fitting into the binding pocket, berberine interacts with CYP1B1 in a more structurally constrained manner. Both ligands demonstrate stable binding, but the slightly higher flexibility of proanthocyanidin may reflect its exploratory binding nature as a novel inhibitor. Together with RMSD and protein RMSF data, these findings further support the potential of proanthocyanidin as a promising CYP1B1 inhibitor, warranting further optimization.

### Radius of gyration (Rg)

**Fig. 9:**
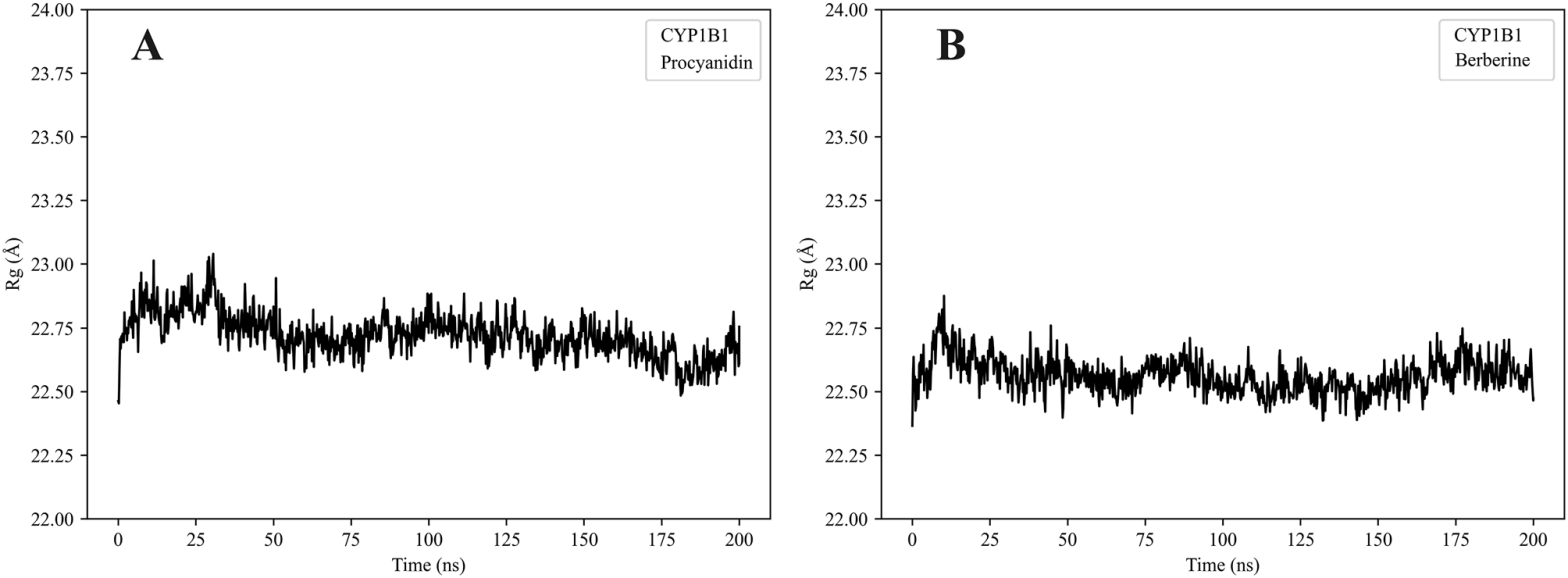
Rg profiles of CYP1B1 in complex with different ligands over 200 ns MD simulations. (**A**) CYP1B1–proanthocyanidin complex; (**B**) CYP1B1–berberine complex.

To assess the overall compactness and structural stability of CYP1B1 in response to ligand binding, the radius of gyration (Rg) was calculated for both the proanthocyanidin- and berberine-bound systems throughout the 200 ns molecular dynamics simulations. As shown in Fig. 9, the Rg values of both complexes remained within a narrow range of 22.5 to 23.0 Å, indicating that the global architecture of the protein was preserved and no significant conformational expansion occurred during the simulation.

Comparative analysis revealed that the berberine-bound CYP1B1 complex exhibited slightly reduced fluctuations in Rg values relative to the proanthocyanidin-bound form, suggesting a marginally more compact conformation. This observation aligns with the RMSF trends and indicates that both ligands maintained the structural integrity of CYP1B1, with berberine exerting a somewhat greater stabilizing effect on the protein framework.

Overall, the sustained stability in Rg across both systems confirms that ligand binding did not disrupt the enzyme’s core structural organization under the simulated conditions, supporting the conformational compatibility of both compounds with the CYP1B1 binding pocket.

### Solvent accessible surface area (SASA)

**Fig. 10:**
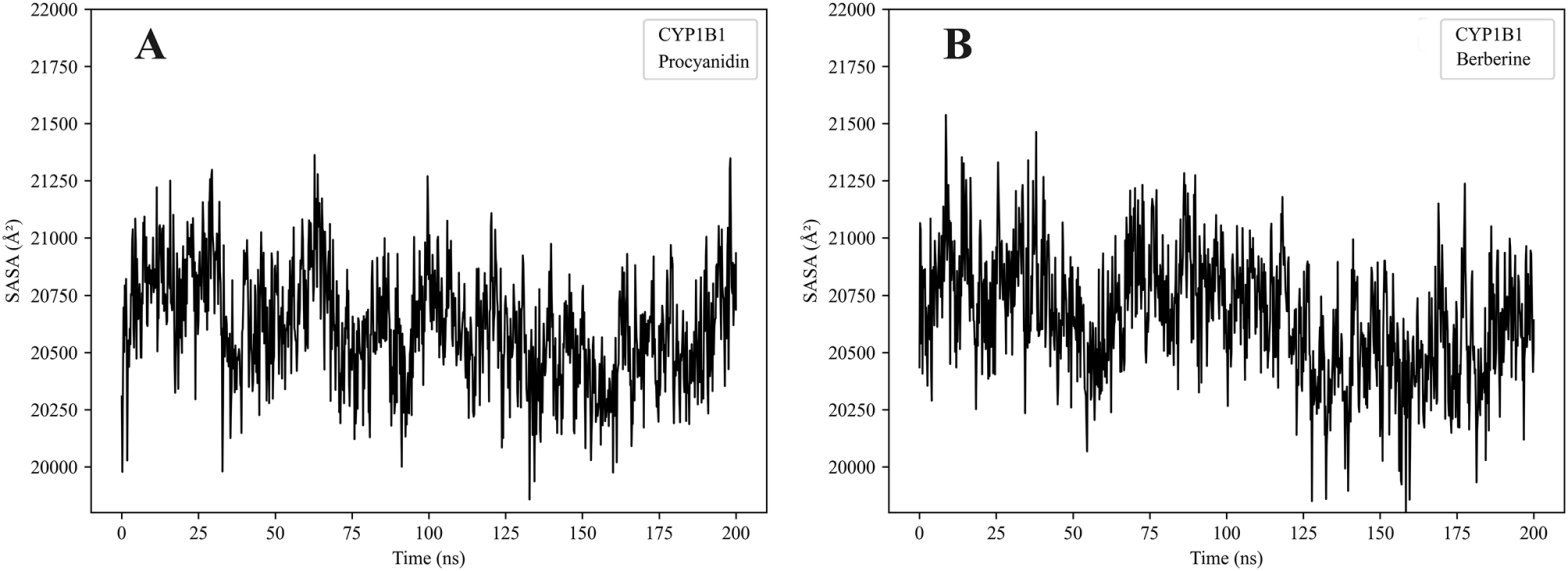
Solvent Accessible Surface Area (SASA) of CYP1B1 in complex with proanthocyanidin (**A**) and berberine (**B**) over a 200 ns molecular dynamics simulation. Both systems exhibit similar fluctuation ranges (20,000–21,800 Å^2^), indicating stable protein surface exposure under simulated conditions.

As shown in Fig. 10, the solvent accessible surface area (SASA) values of CYP1B1 in complex with both proanthocyanidin (A) and berberine (B) remained relatively stable throughout the 200 ns molecular dynamics simulations. The values fluctuated within a narrow range of approximately 20,250 to 21,000 Å^2^, without displaying any significant upward or downward trends.

Both complexes exhibited minor fluctuations in SASA during the early phase of simulation (0–50 ns), likely corresponding to local structural adjustments as the systems reached equilibrium. Beyond this equilibration phase, the SASA curves stabilized with only transient deviations, indicating no major unfolding or conformational disruption occurred during the simulations.

Overall, these results suggest that neither proanthocyanidin nor berberine caused significant changes in the global solvent exposure of CYP1B1. The similar SASA profiles for both complexes reflect a comparable degree of structural integrity and conformational compatibility, supporting the notion that both compounds fit well within the protein’s binding environment without inducing destabilization.

### ADMET prediction of proanthocyanidin

To further evaluate the pharmacokinetic profile and drug-likeness of proanthocyanidin, a set of in silico ADMET predictions was conducted. As shown in Table 1, the compound exhibited high human intestinal absorption (HIA = 0.98), indicating favorable uptake in the gastrointestinal tract. However, the Caco-2 permeability was low (–6.76), suggesting limited passive diffusion across intestinal epithelial barriers.

**Table 1:** ADMET analysis of proanthocyanidin.

Category	Property	Value	Interpretation
Absorption	Human intestinal absorption (HIA)	0.98 (High)	Good predicted intestinal absorption
Absorption	Caco-2 permeability	−6.76 (Low)	Low permeability may limit absorption
Distribution	Blood–brain barrier (BBB) penetration	0.013 (No)	Unlikely to cross blood–brain barrier
Metabolism	CYP3A4 inhibition	0.167 (No)	Low risk of CYP3A4 interaction
Toxicity	AMES toxicity	0.269 (No)	Non-mutagenic
Toxicity	Hepatotoxicity (DILI)	0.253 (Moderate)	Potential for liver injury
Drug-likeness	Lipinski rule of five	Violated (3 violations)	May affect oral bioavailability and drug-likeness

In terms of distribution, proanthocyanidin was not predicted to cross the blood–brain barrier (BBB penetration = 0.013), which may reduce the risk of central nervous system-related side effects. The metabolic liability appears low, as the compound showed no significant inhibition of CYP3A4 (probability = 0.167), indicating a minimal risk of drug–drug interactions mediated by this major enzyme.

Toxicity assessments revealed a non-mutagenic profile (AMES test = 0.269) and moderate risk of hepatotoxicity (DILI probability = 0.253), highlighting the need for further toxicological validation in biological systems. Additionally, proanthocyanidin violated Lipinski’s Rule of Five (three violations) due to its high molecular weight, excessive hydrogen bond donors, and acceptors, which may limit its oral bioavailability and drug-likeness.

Overall, these findings suggest that while proanthocyanidin demonstrates promising safety and absorption characteristics, its poor permeability and physicochemical violations warrant optimization or alternative delivery strategies to enhance its systemic availability and clinical applicability.

## Discussion

Our kinetic analysis demonstrated that proanthocyanidin (PA) is a potent inhibitor of CYP1B1, with a mixed-type mechanism and an IC₅₀ of 2.53 μM. Mixed-type inhibition indicates that PA can bind to both the free enzyme and the enzyme–substrate complex, reducing catalytic efficiency in a manner not strictly competitive. Although the reversibility of inhibition was not experimentally determined, the absence of electrophilic or reactive groups in PA suggests that the inhibition is likely reversible, consistent with the behavior of most polyphenolic CYP inhibitors.

Molecular docking of PA into the crystal structure of CYP1B1 revealed a coherent binding mode that helps explain its inhibitory effects. In the docked pose, PA forms hydrogen bonds with *Asn228* and *Asn265* of CYP1B1, and its polyphenolic ring system engages in π–π stacking with *Phe231* in the enzyme’s active site. These specific contacts align well with known ligand recognition features of CYP1B1. For example, crystallographic and computational studies have shown that the F-helix residue Phe231 in CYP1B1 provides a key aromatic stacking platform for planar inhibit^27^. Likewise, *Asn228* and *Asn265* are frequently observed as anchoring points for inhibitor binding in CYP1B1, often forming hydrogen bonds with ligand substituents^27^. Interestingly, the importance of Asn228 is corroborated by mutagenesis data: substituting Asn228 with Thr in CYP1B1 abolishes berberine’s inhibitory effect^28^ highlighting how critical this residue is for ligand interaction. Our docking results suggest PA exploits the same hot-spot residues (Asn228, Asn265, Phe231) as other inhibitors, reinforcing confidence in the binding model. These interactions are consistent with a non-covalent and likely reversible binding mode. Importantly, no covalent or reactive intermediate structures were predicted, and the chemical structure of PA does not contain electrophilic groups that would typically lead to irreversible enzyme modification or intermediate complex formation.

To further validate the stability of the PA-CYP1B1 complex, we carried out 200 ns molecular dynamics (MD) in aqueous solution. The MD showed a stable root-mean-square deviation (RMSD) for both the protein and PA, with fluctuations plateauing early in the simulation, indicative of a well-accommodated ligand. The radius of gyration (Rg) of CYP1B1 remained essentially constant, suggesting no significant unfolding or collapse of the enzyme upon PA binding. Residue flexibility analysis (RMSF) revealed only localized increases in loop mobility, whereas active-site residues around PA (including Asn228 and Phe231) maintained low flexibility, consistent with a snug fit. Solvent-accessible surface area (SASA) profiles of the protein–ligand complex were also stable over time, implying that PA remained buried in the binding pocket without dissociation. These MD observations substantiate the docking pose by confirming that PA can be stably accommodated in the CYP1B1 active site for an extended period.

To enhance the robustness of our results, a parallel 200 ns molecular dynamics (MD) simulation was conducted using berberine, a well-established CYP1B1 inhibitor, as a positive control. The comparison revealed that both proanthocyanidin and berberine sustained stable conformations within the enzyme’s active site throughout the simulation. Key dynamic parameters, including RMSD, RMSF, Rg, and SASA, exhibited only minor fluctuations in both complexes. This high degree of similarity supports the structural compatibility of proanthocyanidin with the CYP1B1 binding site and further underscores its potential as a stable and effective inhibitor.

To further contextualize PA’s inhibition mechanism, we compared its structural interactions with those of other well-characterized natural CYP1B1 inhibitors. For instance, molecular docking studies have shown that *berberine* and *resveratrol* also interact with key residues such as Phe231 and Asn228, similar to PA. However, unlike these planar monomeric compounds, PA’s polyhydroxylated and oligomeric structure allows for multi-point anchoring within the active site, engaging not only Asn228 and Asn265 through hydrogen bonding but also forming π–π stacking with both Phe231 and the heme group. This extended interaction profile may contribute to its observed mixed-type inhibition.

To better contextualize PA’s potency and inhibition mechanism, we next compared its activity to other naturally occurring CYP1B1 inhibitors. For example, estrone (E1), a steroidal compound, was shown to suppress TCDD-induced CYP1B1 activity in MCF-7 cells with an IC_50_ of 2.5 μM. Estrone is believed to act via a competitive inhibition mechanism, likely due to its structural similarity to endogenous estrogen substrates^29^. In contrast, methoxyflavonoids like 7-methoxyflavone exhibit comparatively weaker inhibition (IC_50_ = 12 μM), and have been reported to inhibit CYP1 enzymes primarily through noncompetitive or uncompetitive modes, possibly by interfering with electron transfer^30^. Notably, homoeriodictyol, a flavanone compound, represents one of the most potent naturally occurring CYP1B1 inhibitors reported to date, with an IC_50_ of 0.24 μM and strong selectivity toward CYP1B1 over CYP1A isoforms^31^. These comparisons illustrate that while many natural CYP1B1 inhibitors act via competitive or noncompetitive mechanisms, PA’s mixed-type inhibition is mechanistically distinct, potentially allowing for dual-site interaction. This places PA among the moderately strong CYP1B1 inhibitors of natural origin and underscores its potential utility in selective CYP1B1-targeted chemoprevention strategies.

ADMET predictions were employed to gauge the drug-like properties of PA and anticipate any potential pharmacokinetic or toxicity liabilities. The ADMET analysis suggested high intestinal absorption for PA, with a Human Intestinal Absorption (HIA) score of 0.98. This implies that, if administered orally, PA has a favorable chance of being absorbed through the gut wall. Paradoxically, the same analysis predicted low Caco-2 cell permeability (− 6.76), which is below the optimal range and would typically signal poor passive diffusion. This discrepancy between high HIA and low Caco-2 permeability could indicate that PA’s absorption might involve active transport mechanisms or metabolism-dependent uptake that is not captured by the Caco-2 model. It may also simply reflect limitations of the predictive models for molecules like PA, which fall outside the typical drug-like chemical space. Consistent with its polyphenolic, high-molecular-weight structure (MW 594), PA did violate multiple drug-likeness rules (e.g. Lipinski’s rule of 5) in our analysis—notably due to a large number of hydrogen bond donors (nHD = 10) and a high polar surface area (~ 230 Å^2^). Such features often correlate with poor membrane permeability and oral bioavailability. Indeed, many proanthocyanidins and related polyphenols are known to have limited bioavailability, especially in their intact form, unless they are small enough or metabolized into absorbable units^32^. This must be kept in mind when considering PA as a lead for drug development.

On a positive note, PA’s predicted metabolic and toxicity profile appears encouraging. Importantly, PA showed minimal predicted inhibition of CYP3A4 (probability ~ 0.17 of being an inhibitor), suggesting a low risk of interfering with the major drug-metabolizing enzyme in humans. It was similarly predicted not to significantly inhibit other CYP isoforms (CYP1A2, 2C9, 2D6, etc.) according to ADMETlab outputs, implying a degree of selectivity for CYP1B1—a desirable trait for avoiding off-target drug-drug interactions. Additionally, PA did not flag strong risks for cardiotoxicity (hERG blocker probability very low) or hepatotoxicity in the in silico toxicity panel, although a high propensity for skin sensitization was noted. Overall, the ADMET predictions paint PA as a compound with high oral absorption potential but formulation challenges, and with a relatively benign safety profile except for its physicochemical unsuitability as a conventional “drug-like” molecule. To overcome these limitations, formulation strategies such as nanoencapsulation, prodrug derivatization, or lipid-based delivery systems may be explored to improve its bioavailability and pharmacokinetic properties. These computational predictions provide early guidance; however, they require experimental validation. The strengths of such in silico ADMET screening lie in swiftly identifying potential liabilities (like poor permeability or toxicity flags) before investing in laborious wet-lab studies. The limitation, of course, is that these are statistical predictions which may not fully capture biological complexity-especially for natural products that may be substrates for gut microbiota or subject to conjugation (glucuronidation, sulfation) affecting their true bioavailability. Therefore, while our ADMET data support the feasibility of PA as a safe chemopreventive agent, they also highlight the need for pro-drug or formulation strategies to improve its pharmacokinetic properties. Given the observed limitations in passive permeability and oral bioavailability revealed by in silico ADMET analysis, formulation-based strategies may enhance the translational potential of PA. Techniques such as nanoparticle encapsulation, liposomal delivery, and prodrug derivatization have shown success in improving the solubility, stability, and absorption of other polyphenolic compounds like curcumin and quercetin. Adapting such approaches for PA could help mitigate its physicochemical constraints while preserving its biological activity, facilitating its potential development as a chemopreventive or adjunctive therapeutic agent. Beyond its molecular interaction characteristics, the biological implications of CYP1B1 inhibition warrant special attention.

The discovery of PA as a CYP1B1 inhibitor is particularly significant in the context of cancer chemoprevention. CYP1B1 is an extrahepatic P450 enzyme that plays a prominent role in the metabolic activation of various procarcinogens (such as polycyclic aromatic hydrocarbons) and the oxidation of endogenous estrogens to potentially carcinogenic metabolites^33^. It is well documented that CYP1B1 is overexpressed in many tumors relative to normal tissue^34^, and its elevated activity has been implicated in tumor initiation and progression. In hormone-related cancers, for instance, CYP1B1-mediated conversion of estradiol to 4-hydroxyestradiol can lead to DNA damage and oncogenesis. By inhibiting CYP1B1, PA could reduce the formation of such genotoxic metabolites, thereby interrupting an early step in carcinogenesis. This mechanism aligns with the broader chemopreventive effects attributed to dietary polyphenols. Indeed, epidemiological and experimental studies have associated consumption of proanthocyanidin-rich foods with lower cancer incidence^32^. Proanthocyanidins (including proanthocyanidins from grape seeds, berries, etc.) are known to exhibit anti-inflammatory, antiproliferative, and pro-apoptotic activities in cancer models^35^. Our findings provide a plausible enzymatic target underpinning these effects.

Potential role in reversing chemoresistance. Overexpression of CYP1B1 in tumors has been linked to resistance to certain chemotherapeutic drugs, partly because CYP1B1 can metabolize (and thus inactivate) these drugs or otherwise enhance survival pathways in cancer cells^36,37^. In this regard, inhibitors of CYP1B1 have attracted interest as adjuvants that could sensitize tumors to chemotherapy without harming normal tissue. Resveratrol and other polyphenols, for example, have been examined for their ability to inhibit CYP1B1 in tumor cells and potentially restore drug sensitivity^36,38^. Our identification of PA as a new CYP1B1 inhibitor with low off-target CYP3A4 activity suggests it could serve as a selective modulator in tumors that highly express CYP1B1. This selectivity is crucial: by targeting an enzyme predominantly expressed in tumor tissue, PA might exert anticancer effects while sparing most normal physiological processes (CYP1B1 is minimally expressed in most normal organs). In sum, the dual implications of PA’s activity—blocking carcinogen activation (preventive) and possibly mitigating drug resistance (therapeutic)—underscore its relevance to cancer prevention and treatment strategies. Given its mixed-type inhibition, PA may be synergistic with competitive CYP1B1 inhibitors or chemotherapeutics metabolized by CYP1B1, such as paclitaxel. Similar synergy has been reported for other polyphenols like resveratrol. This possibility warrants further investigation in future combination studies.

While this study provides compelling in vitro and in silico evidence for PA’s inhibitory effect on CYP1B1, we acknowledge that the lack of validation in cellular or in vivo models represents a limitation. Future studies are warranted to assess the pharmacodynamic efficacy and tissue-selective effects of PA in CYP1B1-overexpressing cancer models. In particular, evaluating PA’s ability to suppress CYP1B1-mediated estrogen metabolism or chemoresistance in breast, ovarian, or endometrial cancer cell lines could offer valuable translational insight. Furthermore, future research should consider exploring the inhibitory effects of other types of proanthocyanidins or related polyphenols, as these compounds, with their structural diversity, may present different levels of potency or selectivity toward CYP1B1. In vivo pharmacokinetic and toxicity profiling will also be crucial to determine its suitability for chemopreventive or adjunctive applications.

## Conclusion

This study establishes a mechanistic basis for CYP1B1 inhibition by proanthocyanidin (PA), highlighting its potential as a natural chemopreventive agent. By integrating enzyme kinetics, molecular docking, molecular dynamics, and ADMET analysis, we showed that PA acts via a mixed-type inhibition mechanism and exhibits favorable in silico safety properties. Nonetheless, limitations such as low predicted permeability, lack of structural validation, and absence of biological efficacy data warrant further investigation. Addressing these gaps through structure–activity refinement, formulation strategies, and in vivo validation will be critical for advancing PA toward therapeutic application. Overall, our findings lay a foundation for the development of PA-based CYP1B1 inhibitors and contribute to the broader effort of harnessing dietary polyphenols in cancer prevention.

## Supplementary Information

Supplementary Video 1.

Supplementary Information 1.

## Data Availability

The datasets generated during the current study are available from the corresponding author upon reasonable request.

## References

[1] Li, F., Zhu, W. & Gonzalez, F. J. Potential role of CYP1B1 in the development and treatment of metabolic diseases. *Pharmacol. Ther.* **178**, 18–30 (2017). 28322972 10.1016/j.pharmthera.2017.03.007 PMC5600638

[2] Kwon, Y.-J., Shin, S. & Chun, Y.-J. Biological roles of cytochrome P450 1A1, 1A2, and 1B1 enzymes. *Arch. Pharmacal. Res.* **44**(1), 63–83 (2021). 10.1007/s12272-021-01306-w 33484438

[3] Hayes, C. L. et al. 17 beta-estradiol hydroxylation catalyzed by human cytochrome P450 1B1. *Proc. Natl. Acad. Sci.* **93**(18), 9776–9781 (1996). 8790407 10.1073/pnas.93.18.9776 PMC38505

[4] Cavalieri, E. L. et al. Molecular origin of cancer: Catechol estrogen-3,4-quinones as endogenous tumor initiators. *Proc. Natl. Acad. Sci.* **94**(20), 10937–10942 (1997). 9380738 10.1073/pnas.94.20.10937 PMC23537

[5] Clemons, M. & Goss, P. Estrogen and the risk of breast cancer. *N. Engl. J. Med.* **344**(4), 276–285 (2001). 11172156 10.1056/NEJM200101253440407

[6] Go, R.-E., Hwang, K.-A. & Choi, K.-C. Cytochrome P450 1 family and cancers. *J. Steroid Biochem. Mol. Biol.* **147**, 24–30 (2015). 25448748 10.1016/j.jsbmb.2014.11.003

[7] Falero-Perez, J. et al. Cyp1b1 expression impacts the angiogenic and inflammatory properties of liver sinusoidal endothelial cells. *PLoS ONE* **13**(10), e0206756 (2018). 30372497 10.1371/journal.pone.0206756 PMC6205649

[8] Berstein, L. M. et al. Aromatase, CYP1B1 and fatty acid synthase expression in breast tumors of BRCA1 mutation carriers. *Pathol. Oncol. Res.* **15**(3), 407–409 (2008). 10.1007/s12253-008-9137-6 19083124

[9] Piccinato, C. A. et al. Increased expression of CYP1A1 and CYP1B1 in ovarian/peritoneal endometriotic lesions. *Reproduction* **151**(6), 683–692 (2016). 27012269 10.1530/REP-15-0581

[10] Sridhar, J. et al. Insights on cytochrome P450 enzymes and inhibitors obtained through QSAR studies. *Molecules* **17**(8), 9283–9305 (2012). 22864238 10.3390/molecules17089283 PMC3666846

[11] McFadyen, M. C. et al. Cytochrome P450 CYP1B1 protein expression. *Biochem. Pharmacol.* **62**(2), 207–212 (2001). 11389879 10.1016/s0006-2952(01)00643-8

[12] Mitsui, Y. et al. CYP1B1 promotes tumorigenesis via altered expression of CDC20 and DAPK1 genes in renal cell carcinoma. *BMC Cancer* **15**(1), 942 (2015). 26626260 10.1186/s12885-015-1951-0 PMC4665921

[13] Rendic, S. & Guengerich, F. P. Contributions of human enzymes in carcinogen metabolism. *Chem. Res. Toxicol.* **25**(7), 1316–1383 (2012). 22531028 10.1021/tx300132k PMC3398241

[14] Luo, B. et al. Cytochrome P450: Implications for human breast cancer (Review). *Oncol. Lett.* **22**(1), 548 (2021). 34093769 10.3892/ol.2021.12809 PMC8170261

[15] Zanger, U. M. & Schwab, M. Cytochrome P450 enzymes in drug metabolism: Regulation of gene expression, enzyme activities, and impact of genetic variation. *Pharmacol. Ther.* **138**(1), 103–141 (2013). 23333322 10.1016/j.pharmthera.2012.12.007

[16] Pandey, K. B. & Rizvi, S. I. Plant polyphenols as dietary antioxidants in human health and disease. *Oxid. Med. Cell. Longev.* **2**(5), 270–278 (2009). 20716914 10.4161/oxim.2.5.9498 PMC2835915

[17] Scalbert, A. et al. Dietary polyphenols and the prevention of diseases. *Crit. Rev. Food Sci. Nutr.* **45**(4), 287–306 (2005). 16047496 10.1080/1040869059096

[18] Yang, C. S., Wang, H. & Sheridan, Z. P. Studies on prevention of obesity, metabolic syndrome, diabetes, cardiovascular diseases and cancer by tea. *J. Food Drug Anal.* **26**(1), 1–13 (2018). 29389543 10.1016/j.jfda.2017.10.010 PMC9332647

[19] Guengerich, F. P. Mechanisms of cytochrome P450-catalyzed oxidations. *ACS Catal.* **8**(12), 10964–10976 (2018). 31105987 10.1021/acscatal.8b03401 PMC6519473

[20] Abotaleb, M. et al. Therapeutic potential of plant phenolic acids in the treatment of cancer. *Biomolecules* **10**(2), 221 (2020). 32028623 10.3390/biom10020221 PMC7072661

[21] Lu, W. et al. Antioxidant activity and healthy benefits of natural pigments in fruits: A review. *Int. J. Mol. Sci.* **22**(9), 4945 (2021). 34066601 10.3390/ijms22094945 PMC8125642

[22] Papavassiliou, K. A., Sofianidi, A. A. & Papavassiliou, A. G. Anthocyanins in non-small cell lung cancer (NSCLC) treatment and prevention. *Nutrients* **16**(10), 1458 (2024). 38794696 10.3390/nu16101458 PMC11124329

[23] Mayer, R. T. B. M. Ethoxyresorufin direct fluorimetric assay of a microsomal O-dealkylation which is preferentially inducible by 3-methylcholanthrene. *Drug Metab. Dispos.* **6**(2), 583–588 (1974). 4155680

[24] Meng, X. et al. A hydroxylated flavonol, fisetin inhibits the formation of a carcinogenic estrogen metabolite. *Steroids* **119**, 53–56 (2017). 28119082 10.1016/j.steroids.2017.01.002

[25] Meng, X. et al. A kaempferol-3-O-β-d-glucoside, intervention effect of astragalin on estradiol metabolism. *Steroids* **149**, 108413 (2019). 31152828 10.1016/j.steroids.2019.05.005

[26] Denisov, I. G. et al. Structure and chemistry of cytochrome P450. *Chem. Rev.* **105**(6), 2253–2278 (2005). 15941214 10.1021/cr0307143

[27] Lee, J.-Y. et al. Adaptable small ligand of CYP1 enzymes for use in understanding the structural features determining isoform selectivity. *ACS Med. Chem. Lett.* **9**(12), 1247–1252 (2018). 30613334 10.1021/acsmedchemlett.8b00409 PMC6295865

[28] Lo, S.-N. et al. Inhibition of CYP1 by berberine, palmatine, and jatrorrhizine: Selectivity, kinetic characterization, and molecular modeling. *Toxicol. Appl. Pharmacol.* **272**(3), 671–680 (2013). 23886934 10.1016/j.taap.2013.07.005

[29] Siddique, M. U. M. et al. Phytoestrogens and their synthetic analogues as substrate mimic inhibitors of CYP1B1. *Eur. J. Med. Chem.* **163**, 28–36 (2019). 30503941 10.1016/j.ejmech.2018.11.039

[30] Takemura, H. et al. Selective inhibition of methoxyflavonoids on human CYP1B1 activity. *Bioorg. Med. Chem.* **18**(17), 6310–6315 (2010). 20696580 10.1016/j.bmc.2010.07.020

[31] Doostdar, H., Burke, M. D. & Mayer, R. T. Bioflavonoids: selective substrates and inhibitors for cytochrome P450 CYP1A and CYP1B1. *Toxicology* **144**(1–3), 31–38 (2000). 10781868 10.1016/s0300-483x(99)00215-2

[32] Nandakumar, V., Singh, T. & Katiyar, S. K. Multi-targeted prevention and therapy of cancer by proanthocyanidins. *Cancer Lett.* **269**(2), 378–387 (2008). 18457915 10.1016/j.canlet.2008.03.049 PMC2562893

[33] Itoh, T. et al. A 3D model of CYP1B1 explains the dominant 4-hydroxylation of Estradiol. *J. Chem. Inf. Model.* **50**(6), 1173–1178 (2010). 20462226 10.1021/ci1000554

[34] Chang, I. et al. Loss of miR-200c up-regulates CYP1B1 and confers docetaxel resistance in renal cell carcinoma. *Oncotarget* **6**(10), 7774–7787 (2015). 25860934 10.18632/oncotarget.3484 PMC4480715

[35] Lee, Y. Cancer chemopreventive potential of procyanidin. *Toxicol. Res.* **33**(4), 273–282 (2017). 29071011 10.5487/TR.2017.33.4.273 PMC5654195

[36] Zhu, Z. et al. CYP1B1 enhances the resistance of epithelial ovarian cancer cells to paclitaxel in vivo and in vitro[J]. *Int. J. Mol. Med.* **35**(2), 340–348 (2014). 25516145 10.3892/ijmm.2014.2041 PMC4292762

[37] Stipp, M. C. et al. Implications of CYP1B1 in the treatment and prognosis of breast cancer. *Beni Suef Univ. J. Basic Appl. Sci.* **14**(1), 22 (2025).

[38] Liu, J., Sridhar, J. & Foroozesh, M. Cytochrome P450 family 1 inhibitors and structure-activity relationships. *Molecules* **18**(12), 14470–14495 (2013). 24287985 10.3390/molecules181214470 PMC4216474

